# Magnetic Resonance Imaging-Defined Cerebral Small Vessel Disease Burden and Longitudinal Neuropsychiatric and Functional Recovery After Stroke: A Prospective Cohort Study

**DOI:** 10.3390/neurolint18090179

**Published:** 2026-09-21

**Authors:** Aishah Ibrahim Albakr, Alia Alokley, Feras Alsulaiman, Abdulrahman Abdulwahab, Mohammad AlWatban, Dalal Albakr, Feras A. Al-Awad, Mustafa Ahmed Alqarni, Saud A. Alnaaim, Ali Hafiz Alhashim, Noman Ishaque, Modhi AlKhaldi, Azra Zafar, Fatimah AlSaihati, Saleh Saad Alotaibi, Firas Saad Alahmari, Norah A. AlKhaldi

**Affiliations:** 1 Department of Neurology, College of Medicine, Imam Abdulrahman Bin Faisal University, Dammam 34212, Saudi Arabia; fasulaiman@iau.edu.sa (F.A.); mqarni@iau.edu.sa (M.A.A.); ahalhashem@iau.edu.sa (A.H.A.); msaalkhaldi@iau.edu.sa (M.A.); azafar@iau.edu.sa (A.Z.); falsaihati@iau.edu.sa (F.A.); 2230600062@iau.edu.sa (S.S.A.); firas-2012@hotmail.com (F.S.A.); nkhaldi@iau.edu.sa (N.A.A.); 2 Clinical Neurosciences Department, College of Medicine, King Faisal University, Ahsa 31982, Saudi Arabia; aalokley@kfu.edu.sa (A.A.);; 3 Department of Radiology, College of Medicine, Imam Abdulrahman Bin Faisal University, Dammam 34212, Saudi Arabia; ahabdulwahab@iau.edu.sa (A.A.); mjwatban@iau.edu.sa (M.A.); 4 Department of Psychiatry, College of Medicine, Imam Abdulrahman Bin Faisal University, Dammam 34212, Saudi Arabia; dmbakr@iau.edu.sa (D.A.); falawad@iau.edu.sa (F.A.A.-A.); 5 Department of Neurology, College of Medicine, University of Saskatchewan, Royal University Hospital, Saskatoon, SK S7N 0W8, Canada; aro890@mail.usask.ca

**Keywords:** cerebral small vessel disease, stroke, post-stroke depression, functional recovery, magnetic resonance imaging

## Abstract

**Background/Objectives:** Cerebral small vessel disease (CSVD) is an established determinant of functional outcome after stroke; however, its influence on the longitudinal course of neuropsychiatric recovery remains incompletely understood. We aimed to investigate the association between MRI-defined CSVD burden and longitudinal depressive symptom trajectories, as well as cognitive, anxiety, and functional outcomes after stroke. **Methods:** This prospective longitudinal cohort study included consecutive patients with acute ischemic stroke or intracerebral hemorrhage treated at a tertiary comprehensive stroke center between January 2022 and November 2025. CSVD burden was quantified using a validated MRI-based Global CSVD Score and categorized as none-to-mild (0–1) or moderate-to-severe (2–4). Depressive symptoms were assessed using the 17-item Hamilton Depression Rating Scale (HDRS-17), anxiety using the Generalized Anxiety Disorder-7 (GAD-7), cognitive function using the Montreal Cognitive Assessment (MoCA), and functional outcome using the modified Rankin Scale (mRS) at baseline and 3, 6, and 9–12 months after stroke. Longitudinal depressive symptom trajectories were evaluated using linear mixed-effects models, and multivariable logistic regression was used to assess associations with incident and persistent post-stroke depression, cognitive impairment, and functional outcome. **Results:** Of 550 participants enrolled, 480 (87.3%) underwent brain MRI and were included in the MRI-defined CSVD analyses. Moderate-to-severe CSVD burden was associated with a less favorable longitudinal depressive symptom trajectory, reflected by a significant CSVD burden × time interaction (β = 0.68, 95% CI 0.25–1.11; *p* = 0.002). Moderate-to-severe CSVD burden was also independently associated with poor functional outcome (adjusted odds ratio [aOR] 1.56, 95% confidence interval [CI] 1.04–2.32; *p* = 0.030). In contrast, baseline CSVD burden was not independently associated with incident post-stroke depression, persistent post-stroke depression, or global cognitive impairment after multivariable adjustment. **Conclusions:** Greater MRI-defined CSVD burden was associated with a less favorable longitudinal depressive symptom trajectory and poorer functional recovery after stroke, whereas it was not independently associated with incident or persistent post-stroke depression or global cognitive impairment. These findings suggest that cumulative MRI-defined CSVD burden may provide complementary information regarding longitudinal emotional and functional recovery, although its incremental prognostic value and clinical utility require further validation. Multicenter studies with longer follow-up and quantitative neuroimaging are needed to confirm these associations and determine whether CSVD assessment improves risk stratification and individualized post-stroke monitoring and rehabilitation.

## 1. Introduction

Advances in acute stroke care have substantially reduced mortality, resulting in a growing population of stroke survivors living with long-term neurological and neuropsychiatric sequelae [1]. Post-stroke depression (PSD), cognitive impairment, and other neuropsychiatric complications are increasingly recognized as major contributors to long-term disability, reduced quality of life, impaired social reintegration, and caregiver burden [2,3,4,5]. Although acute stroke management has traditionally focused on motor and sensory deficits, cognitive and psychological sequelae may exert an equal or greater influence on long-term independence, participation, and quality of life [2,3,4]. PSD is among the most common neuropsychiatric complications of stroke, affecting approximately one-third of survivors within the first year [5]. Importantly, neuropsychiatric outcomes are heterogeneous and may follow acute, delayed-onset, recurrent, or persistent courses over time [5,6,7]. Studies from Saudi Arabia have similarly demonstrated a substantial burden of PSD, underscoring the importance of identifying factors associated with neuropsychiatric recovery in this population [8].

Emotional and cognitive impairments frequently coexist and are not fully explained by conventional measures of stroke severity or lesion location. Patients with relatively mild neurological deficits may experience substantial depression, anxiety, cognitive impairment, and reduced quality of life despite good neurological recovery [3,7,9,10,11]. These findings suggest that factors beyond the characteristics of the index stroke may contribute to long-term recovery and highlight the need to better understand the biological factors associated with neuropsychiatric outcomes after stroke.

Cerebral small vessel disease (CSVD) is increasingly recognized as an important contributor to stroke outcomes, vascular cognitive impairment, functional disability, and neuropsychiatric symptoms worldwide [12,13]. CSVD comprises a spectrum of pathological changes affecting cerebral arterioles, capillaries, and venules and is typically characterized on neuroimaging by white matter hyperintensities, lacunes, cerebral microbleeds, and enlarged perivascular spaces [12,14,15,16]. Unlike large-vessel cerebrovascular disease, CSVD can disrupt widespread brain networks through cumulative white matter injury, subcortical disconnection, impaired neurovascular function, and loss of structural connectivity [12,16,17,18]. Consequently, CSVD is increasingly regarded as a diffuse cerebrovascular disorder affecting distributed brain networks and reflecting cumulative brain injury rather than merely an incidental neuroimaging finding [16,17,18,19,20].

CSVD contributes to cognitive decline, functional disability, depression, and other neuropsychiatric manifestations. Several studies have reported associations between MRI-defined CSVD burden and depressive symptoms after stroke, while network-based investigations have linked white matter disruption and impaired structural connectivity with both depression and cognitive impairment in patients with CSVD [21,22,23,24,25,26]. However, most previous studies have focused on individual CSVD markers, cross-sectional associations, or isolated clinical outcomes rather than examining the relationship between cumulative MRI-defined CSVD burden and longitudinal neuropsychiatric and functional recovery [22,23,24,26,27]. Longitudinal assessment may therefore provide greater insight into the clinical relevance of cumulative CSVD burden and its association with post-stroke recovery trajectories.

This study aimed to investigate the association between cumulative MRI-defined CSVD burden and longitudinal neuropsychiatric and functional recovery following acute stroke. We hypothesized that greater CSVD burden would be associated with a less favorable longitudinal depressive symptom trajectory and poorer functional recovery. Furthermore, we explored whether baseline stroke severity modified the association between CSVD burden and post-stroke depressive outcomes. Secondary analyses evaluated the associations of CSVD burden with anxiety symptoms and global cognitive impairment.

## 2. Materials and Methods

### 2.1. Design and Analytical Strategy

This prospective longitudinal cohort study consecutively enrolled adults (≥18 years) presenting with acute cerebrovascular events to the Comprehensive Stroke Center at King Fahad Hospital of the University, Eastern Region, Saudi Arabia, between January 2022 and November 2025. For the present analysis, eligible participants were those with clinically and radiologically confirmed acute ischemic stroke or intracerebral hemorrhage who fulfilled the study eligibility criteria and had the data required for the relevant outcome analyses.

Antidepressant treatment data were collected as part of the broader prospective cohort and have been examined separately in an analysis focused specifically on real-world antidepressant treatment pathways and outcomes after post-stroke depression. In the present study, these data were used only to address potential treatment-related confounding by incorporating documented antidepressant initiation before each assessment as a time-varying covariate in a sensitivity analysis. Treatment-pathway completion and antidepressant effectiveness were not evaluated as outcomes in the present analysis.

The original study protocol was approved by the Standing Committee for Research Ethics on Living Creatures at Imam Abdulrahman Bin Faisal University (approval No. 2017-200-Med-NF). A revised protocol was subsequently approved by the Institutional Review Board on 16 February 2023 (IRB No. IRB-2023-01-078). The protocol was further amended and the study period extended under the same IRB approval on 30 April 2026. Written informed consent was obtained from all participants or their legally authorized representatives before enrollment. The study was conducted in accordance with the Declaration of Helsinki and is reported in accordance with the Strengthening the Reporting of Observational Studies in Epidemiology (STROBE) guidelines [28].

A predefined analytical framework specified that CSVD-specific analyses would be restricted to participants who underwent brain MRI. MRI acquisition was determined by clinical indication and availability rather than mandated uniformly by the research protocol; consequently, the MRI-defined CSVD cohort may have differed systematically from participants who did not undergo MRI. This potential source of selection bias was considered in the interpretation of the findings and was further evaluated by comparing baseline characteristics according to MRI availability.

### 2.2. Participants

Patients were eligible if they were aged ≥18 years and had a clinically and radiologically confirmed diagnosis of acute ischemic stroke or intracerebral hemorrhage. Participants were required to have sufficient baseline demographic and clinical information and adequate follow-up for the relevant outcome analyses. Brain MRI was performed as part of routine clinical care when clinically indicated and available.

Patients were excluded if they had missing baseline data or insufficient follow-up for outcome assessment. Additional exclusion criteria included global aphasia, impaired consciousness, severe medical illness, cognitive impairment precluding reliable neuropsychiatric assessment, non-vascular intracranial disorders including brain tumors and traumatic brain injury, expected survival of <3 months, or inability to complete assessments in Arabic or English.

### 2.3. Neuroimaging Assessment and CSVD Evaluation

All participants underwent baseline non-contrast head computed tomography as part of routine acute stroke evaluation. Brain MRI was performed in a subset of participants using a 1.5-T Optima MRI system (GE Healthcare, Milwaukee, WI, USA) according to the institutional stroke imaging protocol when clinically indicated and available. The MRI protocol included diffusion-weighted imaging, fluid-attenuated inversion recovery, T2-weighted imaging, and susceptibility-sensitive sequences, including susceptibility-weighted imaging or gradient-recalled echo. CSVD markers were assessed according to the Standards for Reporting Vascular Changes on Neuroimaging (STRIVE) criteria [29,30].

Neuroimaging studies were independently evaluated by two assessors: a neurology resident and a consultant neuroradiologist. Both assessors were blinded to participants’ clinical characteristics, neuropsychiatric and cognitive assessments, and longitudinal functional outcomes. CSVD markers and Global CSVD Scores were assigned according to predefined imaging criteria without knowledge of follow-up outcomes. Discrepancies were resolved by consensus, with final adjudication by the consultant neuroradiologist. Formal inter-rater reliability coefficients were not prospectively calculated; therefore, quantitative inter-rater agreement could not be reported. All MRI examinations included in the CSVD analyses were acquired at 1.5 T, providing consistency in MRI field strength across participants.

### 2.4. Outcomes and Follow-Up

The prespecified outcome measures included incident post-stroke depression (PSD), persistent post-stroke depression, longitudinal depressive symptom trajectories, anxiety symptoms, cognitive impairment, neurological recovery, and functional outcome.

Baseline assessments were completed during the index stroke admission. Follow-up assessments were scheduled at approximately 3 months, 6 months, and 9–12 months after stroke onset. For inclusion in the longitudinal analyses, participants were required to have completed the baseline and 3-month assessments and at least one additional follow-up assessment at either 6 months or 9–12 months. Because not all participants attended every scheduled visit, the longitudinal dataset was unbalanced, with some participants contributing data at 6 months, others at 9–12 months, and some at both time points. Mixed-effects models incorporated all available repeated measurements from eligible participants.

Time was entered as an ordinal numeric variable representing scheduled visit sequence (baseline = 0, 3 months = 1, 6 months = 2, and 9–12 months = 3), thereby estimating the average linear change across successive scheduled study visits. The 9–12 month assessment was treated as a single final scheduled follow-up category rather than according to the exact elapsed time from stroke onset; therefore, participants assessed at 9, 10, 11, or 12 months contributed to the same final follow-up time point.

At each follow-up visit, participants underwent standardized assessment of depressive symptoms, anxiety symptoms, cognitive function, neurological impairment, and functional outcome using the 17-item Hamilton Depression Rating Scale (HDRS-17), Generalized Anxiety Disorder-7 (GAD-7), Montreal Cognitive Assessment (MoCA), National Institutes of Health Stroke Scale (NIHSS), and modified Rankin Scale (mRS), respectively. Follow-up neuropsychiatric and cognitive evaluations were conducted face to face in the outpatient stroke and neurology clinics as structured research assessments.

Neuropsychiatric and cognitive assessments were performed using validated Arabic and English versions of the GAD-7 [31,32], HDRS-17 [33,34,35], and MoCA [36,37,38], according to each participant’s primary language and level of proficiency.

### 2.5. Operational Definitions

The primary exposure was cumulative MRI-defined cerebral small vessel disease (CSVD) burden, quantified using the validated Global CSVD Score according to STRIVE criteria [14,15,30]. White matter hyperintensities were graded using the Fazekas scale [39]. Among participants who underwent MRI, the Global CSVD Score ranged from 0 to 4, with one point assigned for each of the following markers: lacunes, cerebral microbleeds, moderate-to-severe white matter hyperintensities, and moderate-to-severe enlarged perivascular spaces [14].

CSVD burden was analyzed both as a continuous score and categorically. For categorical analyses, Global CSVD Scores of 0–1 were classified as zero-to-mild burden and scores of 2–4 as moderate-to-severe burden. This dichotomization was used to distinguish participants with limited versus multiple MRI-defined CSVD markers and is consistent with previous studies using total CSVD burden scores and similar categorical approaches [14,40,41].

Pre-existing depression was defined as a documented clinical diagnosis of depression before the index stroke or use of antidepressant medication prescribed specifically for depression before stroke onset, consistent with previous stroke cohort studies [42]. Antidepressants prescribed for alternative indications, including neuropathic pain, sensory symptoms, sleep disturbance, or other non-psychiatric conditions, were not considered evidence of pre-existing depression.

The primary outcome was new-onset post-stroke depression (PSD), defined as clinically significant depressive symptoms developing during the follow-up period among participants without pre-existing depression. Depression was assessed using the 17-item Hamilton Depression Rating Scale (HDRS-17), a validated clinician-administered measure of depressive symptom severity [33,35,43]. PSD diagnosis was established through face-to-face clinical assessment by a neurologist or psychiatrist according to Diagnostic and Statistical Manual of Mental Disorders, Fifth Edition (DSM-5) criteria [44], with the HDRS-17 used to quantify symptom severity. An HDRS-17 score ≥ 10 was considered indicative of clinically significant depressive symptoms, consistent with previous post-stroke depression studies [45]. Depression severity was categorized as absent or minimal (<8), mild (8–16), moderate (17–23), or severe (>24) [34].

Secondary outcomes included persistent PSD, longitudinal depressive symptom trajectories, anxiety symptoms, cognitive impairment, neurological recovery, and functional outcome. Persistent PSD was defined as clinically significant depressive symptoms (HDRS-17 ≥ 10) documented at two or more follow-up assessments. Longitudinal depressive symptom trajectories were evaluated using repeated HDRS-17 measurements obtained at the scheduled follow-up visits [33,43,45].

Anxiety symptoms were assessed face to face using the Generalized Anxiety Disorder-7 (GAD-7), supplemented by clinical evaluation [31,32]. A GAD-7 score ≥ 10 was used to define clinically significant anxiety, consistent with the original validation study and previous stroke research [31,46].

Cognitive function was assessed using the Montreal Cognitive Assessment (MoCA) [36,38]. One additional point was added for participants with ≤12 years of formal education, in accordance with standard MoCA administration procedures [36]. Cognitive impairment was defined a priori as a MoCA score < 26. Cognitive status was categorized as normal (≥26), mild impairment (18–25), moderate impairment (10–17), or severe impairment (<10).

Neurological impairment and recovery were assessed using the National Institutes of Health Stroke Scale (NIHSS), with longitudinal change evaluated across scheduled follow-up assessments. Functional outcome was assessed using the modified Rankin Scale (mRS), with poor functional outcome defined a priori as mRS > 2 (mRS 3–6) [47].

For regression analyses using a single follow-up outcome, the assessment time point was prespecified and reported explicitly for each model. Functional outcome analyses used the 3-month (approximately 90-day) mRS assessment, whereas longitudinal depressive, anxiety, cognitive, and neurological outcomes were analyzed using the repeated scheduled assessments described above.

### 2.6. Baseline Variables and Covariates

As this was a prospective observational cohort study, the sample size was determined by the number of eligible participants recruited during the study period. No formal a priori sample-size calculation was performed. The events-per-variable ratio exceeded 10 in the primary multivariable regression models, supporting model stability and reducing the risk of overfitting.

Baseline assessments were performed during the index stroke admission and included demographic, clinical, neuropsychiatric, cognitive, and functional variables. Demographic variables comprised age, sex, educational attainment, and socioeconomic status. Educational attainment was categorized as ≥12 years or <12 years of formal education and was included as a prespecified covariate because of its established association with post-stroke cognitive, functional, and neuropsychiatric outcomes.

Clinical variables included baseline and follow-up National Institutes of Health Stroke Scale (NIHSS) scores, modified Rankin Scale (mRS) scores, vascular risk burden, stroke type, and lesion characteristics. Stroke type was categorized as ischemic stroke or intracerebral hemorrhage. Among participants with ischemic stroke, etiologic categories were assigned based on clinical, vascular, cardiac, and neuroimaging evaluation performed during the index admission and included large-artery atherosclerosis, small-vessel disease, cardioembolic stroke, embolic stroke of undetermined source (ESUS), and other determined causes. Conventional ischemic stroke subtypes were classified according to the TOAST framework [48].

To reduce model complexity and minimize multicollinearity, individual vascular risk factors were combined into a composite vascular risk burden score comprising hypertension, diabetes mellitus, dyslipidemia, current smoking, atrial fibrillation, and prior cerebrovascular disease. Each factor contributed one point, yielding a total score ranging from 0 to 6. For multivariable analyses, vascular risk burden was categorized as low (0–1 risk factors) or moderate-to-high (≥2 risk factors), consistent with the prespecified regression models. Baseline stroke severity was assessed using the NIHSS and modeled as a continuous variable [49,50]. Potential nonlinear associations between stroke severity and study outcomes were explored by additionally including a quadratic NIHSS term (NIHSS^2^).

Lesions were classified as deep or non-deep according to neuroanatomical location. Deep lesions involved the basal ganglia, thalamus, internal capsule, or brainstem, whereas non-deep lesions included cortical and cerebellar infarcts or hemorrhages. Lesion location was included as a prespecified covariate because of its established association with post-stroke neuropsychiatric and functional outcomes [51,52,53,54].

Information on antidepressant initiation and treatment timing was obtained from longitudinal medication records. At each scheduled assessment, participants were classified according to whether antidepressant therapy had been initiated before that assessment. Because detailed information on treatment adherence, medication switching, dose modification, and continuous treatment exposure was not consistently available at every assessment, this variable reflected documented antidepressant initiation before assessment rather than confirmed continuous treatment exposure. Documented antidepressant initiation was subsequently incorporated as a time-varying covariate in the sensitivity analysis, as described in the Section 2.8.

All participants received standard multidisciplinary stroke care, including physiotherapy and rehabilitation according to clinical need, and rehabilitation status was documented during follow-up. However, detailed information regarding the frequency, duration, and intensity of individual rehabilitation sessions was not systematically available and therefore could not be incorporated quantitatively into the longitudinal models.

### 2.7. Outcomes

The primary outcome was incident post-stroke depression. Secondary outcomes included persistent post-stroke depression, longitudinal depressive symptom trajectories, anxiety symptoms, cognitive impairment, neurological recovery, and functional outcome. Detailed operational definitions of all outcome measures are provided in Section 2.5.

For analyses based on a single follow-up assessment, the relevant outcome time point was prespecified for each model. Functional outcome was evaluated using the 3-month (approximately 90-day) mRS assessment. Longitudinal depressive, anxiety, cognitive, neurological, and functional measures were otherwise evaluated using the repeated scheduled assessments at 3 months, 6 months, and 9–12 months, as applicable.

### 2.8. Statistical Analysis

Statistical analyses were performed using IBM SPSS Statistics for Windows, version 26.0 (IBM Corp., Armonk, NY, USA). All statistical tests were two-sided. A *p* value < 0.05 was considered statistically significant for prespecified primary analyses. Multiplicity adjustment was applied to the analyses of individual MRI-defined CSVD markers, whereas exploratory interaction findings were interpreted cautiously as hypothesis-generating.

Baseline demographic, clinical, neuroimaging, cognitive, and neuropsychiatric characteristics were summarized according to MRI-defined CSVD burden category. Continuous variables were assessed for normality using visual inspection of histograms and the Shapiro–Wilk test and are presented as mean ± standard deviation (SD) or median (interquartile range [IQR]), as appropriate. Categorical variables are presented as frequencies and percentages. Between-group comparisons were performed using the independent-samples *t* test or Mann–Whitney U test for continuous variables and the chi-square test or Fisher’s exact test for categorical variables, as appropriate. Effect sizes are reported as Cohen’s d for continuous variables, the phi coefficient for binary categorical variables, and Cramér’s V for categorical variables with more than two categories.

Multivariable logistic regression analyses were performed using complete-case analysis; participants with missing outcome or covariate data were excluded from the corresponding models. Covariates were selected a priori on the basis of clinical relevance and published evidence rather than automated variable-selection procedures. Prespecified covariates included age, sex, educational attainment (<12 vs. ≥12 years), baseline National Institutes of Health Stroke Scale (NIHSS) score, vascular risk burden category, stroke type (ischemic stroke vs. intracerebral hemorrhage), and lesion location. Models evaluating persistent post-stroke depression were additionally adjusted for baseline Hamilton Depression Rating Scale (HDRS-17) score and baseline anxiety symptoms. To minimize multicollinearity, the prespecified composite vascular risk burden category was used in place of individual vascular risk factors. Multicollinearity was assessed using variance inflation factors (VIFs). Model calibration was evaluated using the Hosmer–Lemeshow goodness-of-fit test, and discrimination was assessed using the area under the receiver operating characteristic curve (AUC). Results are presented as adjusted odds ratios (aORs) with corresponding 95% confidence intervals (CIs).

The outcome assessment time point was prespecified for each logistic regression model. Functional outcome models used the 3-month (approximately 90-day) mRS assessment. For depression, anxiety, and cognitive outcomes, the corresponding prespecified follow-up assessment used in each model is reported with the relevant analysis and table. Persistent PSD was defined on the basis of repeated follow-up assessments, as described in Section 2.5.

Potential nonlinear associations between baseline stroke severity and study outcomes were explored using linear and quadratic NIHSS terms (NIHSS and NIHSS^2^). Analyses of individual MRI-defined CSVD markers and interaction effects were considered exploratory. For the four individual CSVD markers—lacunes, cerebral microbleeds, moderate-to-severe white matter hyperintensities, and moderate-to-severe enlarged perivascular spaces—a Bonferroni-adjusted significance threshold of *p* < 0.0125 was applied. Findings from these marker analyses that reached nominal statistical significance but did not remain significant after multiplicity correction were interpreted as hypothesis-generating rather than confirmatory. Likewise, exploratory interaction findings were interpreted cautiously as hypothesis-generating.

Longitudinal changes in depressive symptom severity were analyzed using linear mixed-effects models with participant-specific random intercepts to account for within-participant correlation across repeated HDRS-17 measurements. Fixed effects included follow-up time, baseline CSVD burden category (moderate-to-severe vs. zero-to-mild), and the interaction between follow-up time and CSVD burden. Age, baseline NIHSS score, and baseline HDRS-17 score were included as prespecified covariates. Follow-up time was entered as an ordinal numeric variable representing scheduled visit sequence (baseline = 0, 3 months = 1, 6 months = 2, and 9–12 months = 3), thereby estimating the average linear change across successive scheduled study visits. The 9–12-month assessment was treated as a single final scheduled follow-up category rather than according to the exact elapsed time from stroke onset. The models incorporated all available longitudinal observations and accommodated unbalanced repeated measurements under the assumption that data were missing at random. Model estimates are reported as β coefficients with corresponding 95% CIs.

To evaluate the potential influence of antidepressant treatment on depressive symptom trajectories, the linear mixed-effects model was repeated with documented antidepressant initiation before assessment was included as a time-varying covariate. At each scheduled HDRS-17 assessment, participants were classified as unexposed before documented antidepressant initiation and as exposed at subsequent assessments after initiation. The sensitivity model retained the same fixed effects, interaction terms, prespecified covariates, and participant-specific random-intercept structure as the main longitudinal model. Because detailed information regarding treatment adherence, medication switching, dose modification, and continuous treatment exposure was not consistently available at every assessment, this variable represented documented antidepressant initiation rather than confirmed continuous pharmacological exposure. Because antidepressant treatment was clinically assigned rather than randomized, this sensitivity analysis was used to assess the robustness of the association between CSVD burden and depressive symptom trajectories, and was not interpreted as estimating the causal effect of antidepressant therapy.

Patterns of missing data were evaluated before analysis. No variable included in the primary analyses had substantial missingness requiring imputation. Complete-case analysis was used for the multivariable logistic regression models, whereas the linear mixed-effects models incorporated all available repeated observations under the missing-at-random assumption. Follow-up patterns were unbalanced, with some participants completing the 6-month assessment, others the 9–12-month assessment, and some completing both; therefore, complete follow-up at every scheduled visit was not required for inclusion in the longitudinal models.

To evaluate potential MRI-selection bias, baseline demographic and clinical characteristics were compared between participants who underwent brain MRI and participants who did not undergo MRI, using standardized mean differences (SMDs) and appropriate univariable statistical tests (Supplementary Table S1).

## 3. Results

### 3.1. Study Cohort and Baseline Characteristics

**Figure 1:**
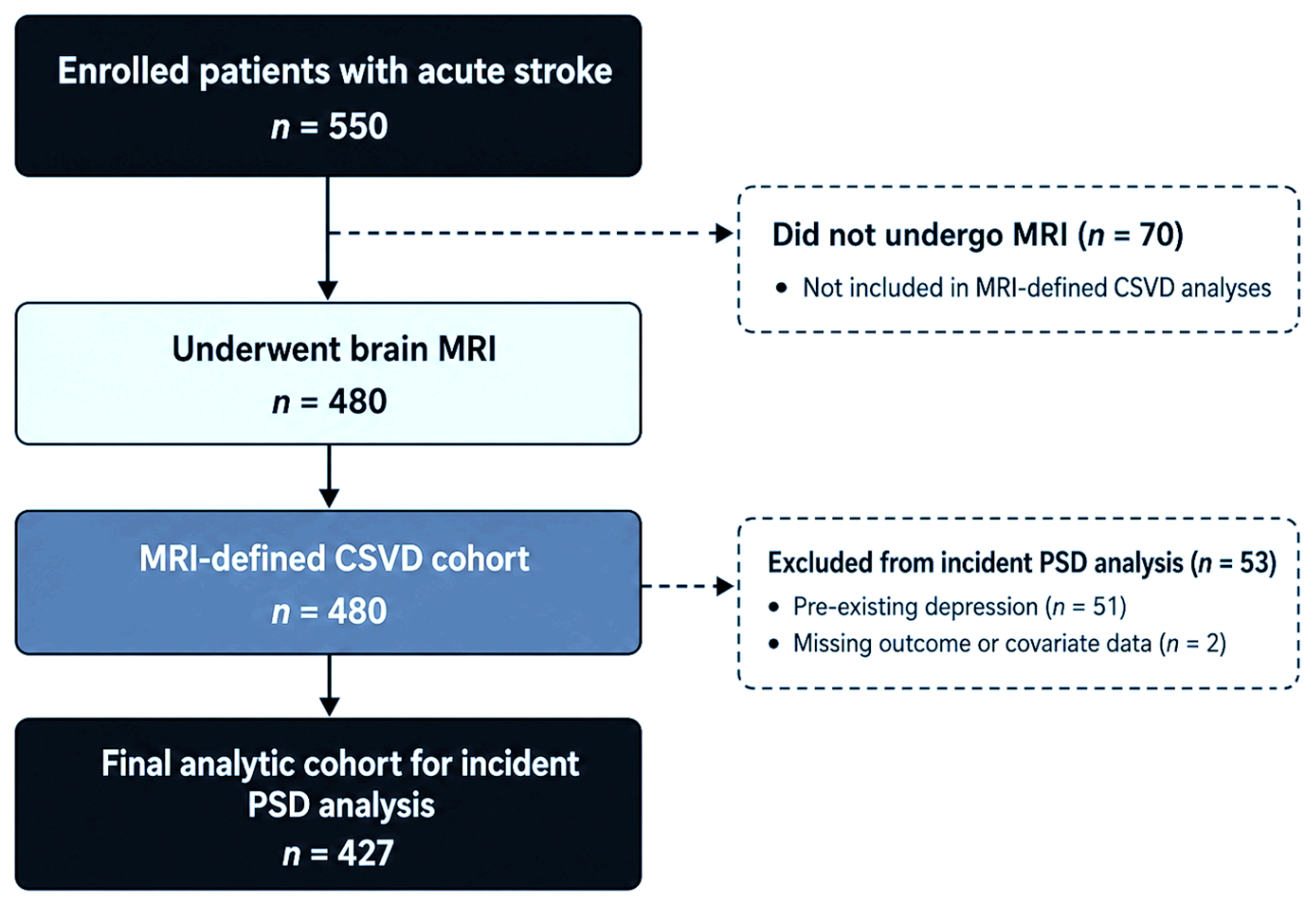
**Flowchart of participant selection and derivation of the MRI-defined cerebral small vessel disease (CSVD) cohort**. The flow diagram illustrates study enrollment, brain MRI availability, derivation of the MRI-defined CSVD cohort, and the final analytical sample for the incident post-stroke depression analysis. Of the 550 enrolled participants with acute stroke, 480 underwent brain MRI and were included in the MRI-defined CSVD cohort, whereas 70 did not undergo MRI. For the incident post-stroke depression analysis, 51 participants with pre-existing depression and 2 with missing outcome or covariate data were excluded, yielding a final analytical sample of 427 participants.

A total of 550 consecutive patients with acute stroke were enrolled during the study period. Of these, 480 (87.3%) underwent brain MRI and comprised the MRI-defined CSVD analytical cohort, whereas 70 (12.7%) did not undergo MRI, as illustrated in Figure 1. Among participants who underwent MRI, the Global CSVD Score was 0 in 55 (11.5%), 1 in 150 (31.3%), 2 in 156 (32.5%), 3 in 92 (19.2%), and 4 in 27 (5.6%).

Baseline demographic and clinical characteristics were generally comparable between participants who underwent MRI and those who did not, with small SMDs for most variables; baseline NIHSS showed a modest imbalance (SMD = 0.21) (Supplementary Table S1).

**Figure 2:**
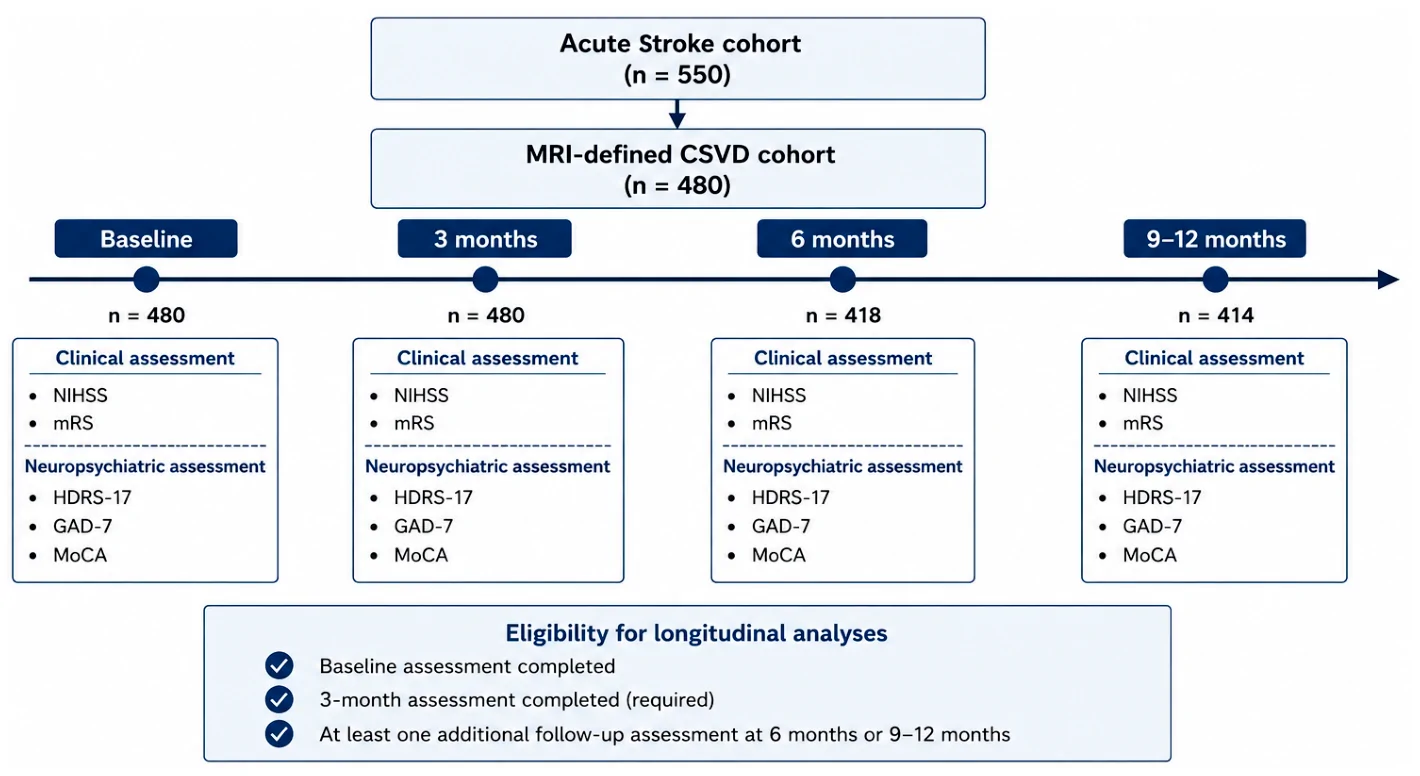
**Longitudinal study design, follow-up schedule, and analytical framework.** The figure illustrates the longitudinal follow-up of the MRI-defined cerebral small vessel disease (CSVD) cohort. Clinical and neuropsychiatric assessments were performed at baseline and at approximately 3, 6, and 9–12 months after stroke. The MRI-defined CSVD cohort included 480 participants at baseline and 3 months, 418 participants at 6 months, and 414 participants at 9–12 months. Participants were eligible for the longitudinal analyses if they completed the baseline and 3-month assessments and at least one additional follow-up assessment at either 6 months or 9–12 months. Linear mixed-effects models incorporated all available repeated observations from eligible participants. Follow-up time was entered as an ordinal numeric variable representing scheduled visit sequence (baseline = 0, 3 months = 1, 6 months = 2, and 9–12 months = 3), with the 9–12-month assessment treated as the final scheduled follow-up category. Depressive symptom severity, assessed using the HDRS-17, was the principal longitudinal neuropsychiatric outcome, whereas anxiety symptoms assessed using the GAD-7 and cognitive performance assessed using the MoCA were evaluated as secondary outcomes. Neurological impairment and functional outcome were assessed using the NIHSS and mRS, respectively. Abbreviations: CSVD, cerebral small vessel disease; GAD-7, 7-item Generalized Anxiety Disorder scale; HDRS-17, 17-item Hamilton Depression Rating Scale; MoCA, Montreal Cognitive Assessment; mRS, modified Rankin Scale; NIHSS, National Institutes of Health Stroke Scale.

Within the MRI-defined CSVD cohort, all 480 participants completed the baseline and 3-month assessments. Follow-up data were available for 418 participants at 6 months and 414 participants at 9–12 months. Because attendance at later follow-up visits was not complete for all participants, the longitudinal dataset was unbalanced. Participants were eligible for the longitudinal mixed-effects analyses if they completed baseline and 3-month assessments and at least one additional assessment at either 6 months or 9–12 months, as shown in Figure 2. Mixed-effects models incorporated all available repeated observations from eligible participants.

For analyses of incident post-stroke depression, 51 participants with documented pre-existing depression were excluded. Two additional participants with missing outcome or covariate data were excluded from the complete-case multivariable analysis, resulting in a final analytical sample of 427 participants (Figure 1). Follow-up patterns were unbalanced. Among the MRI-defined CSVD cohort, 184 participants completed the 3- and 6-month assessments, 151 completed the 3- and 9–12-month assessments, and 215 completed all scheduled follow-up visits; these categories were not mutually exclusive.

Table 1 summarizes baseline characteristics according to MRI-defined CSVD burden. Participants with moderate-to-severe CSVD burden were older than those with zero-to-mild CSVD burden (63.8 ± 13.0 vs. 54.1 ± 14.3 years; *p* < 0.001), had a higher prevalence of hypertension (81.1% vs. 53.2%; *p* < 0.001) and more frequently had a moderate-to-high vascular risk burden (70.9% vs. 58.0%; *p =* 0.005). No significant between-group differences were observed in sex, educational attainment, socioeconomic status, history of depression, baseline NIHSS score, diabetes mellitus, dyslipidemia, smoking, atrial fibrillation, prior stroke/TIA, stroke type, ischemic stroke subtype, or deep lesion involvement.

### 3.2. Longitudinal Depressive Symptom Trajectories According to CSVD Burden

Results of the longitudinal linear mixed-effects model are presented in Table 2. Follow-up time was significantly associated with HDRS-17 scores (β = 1.38, 95% CI [1.06–1.70]; *p* < 0.001). Moderate-to-severe CSVD burden was not independently associated with HDRS-17 scores as a main effect (β = −0.13, 95% CI [−1.01–0.75]; *p* = 0.778). However, a significant CSVD burden × time interaction was observed (β = 0.68; 95% CI [0.25–1.11]; *p* = 0.002), indicating that longitudinal changes in depressive symptom severity differed according to baseline CSVD burden, with a less favorable trajectory among participants with moderate-to-severe CSVD burden.

Baseline NIHSS score (β = 0.23, 95% CI [0.17–0.29]; *p* < 0.001) and baseline HDRS-17 score (β = 0.42, 95% CI [0.32–0.51]; *p* < 0.001) were independently associated with higher longitudinal HDRS-17 scores, whereas age was not significantly associated with HDRS-17 scores (β = 0.01, 95% CI [−0.01–0.04]; *p* = 0.209).

In the sensitivity analysis incorporating documented antidepressant initiation before assessment as a time-varying covariate, the CSVD burden × follow-up time interaction remained statistically significant (β = 0.60; 95% CI [0.17–1.02]; *p* = 0.006), compared with β = 0.68 (95% CI [0.25–1.11]; *p* = 0.002) in the primary model. Therefore, adjustment for documented antidepressant initiation did not materially alter the association between CSVD burden and longitudinal depressive symptom trajectory (Supplementary Table S4).

**Figure 3:**
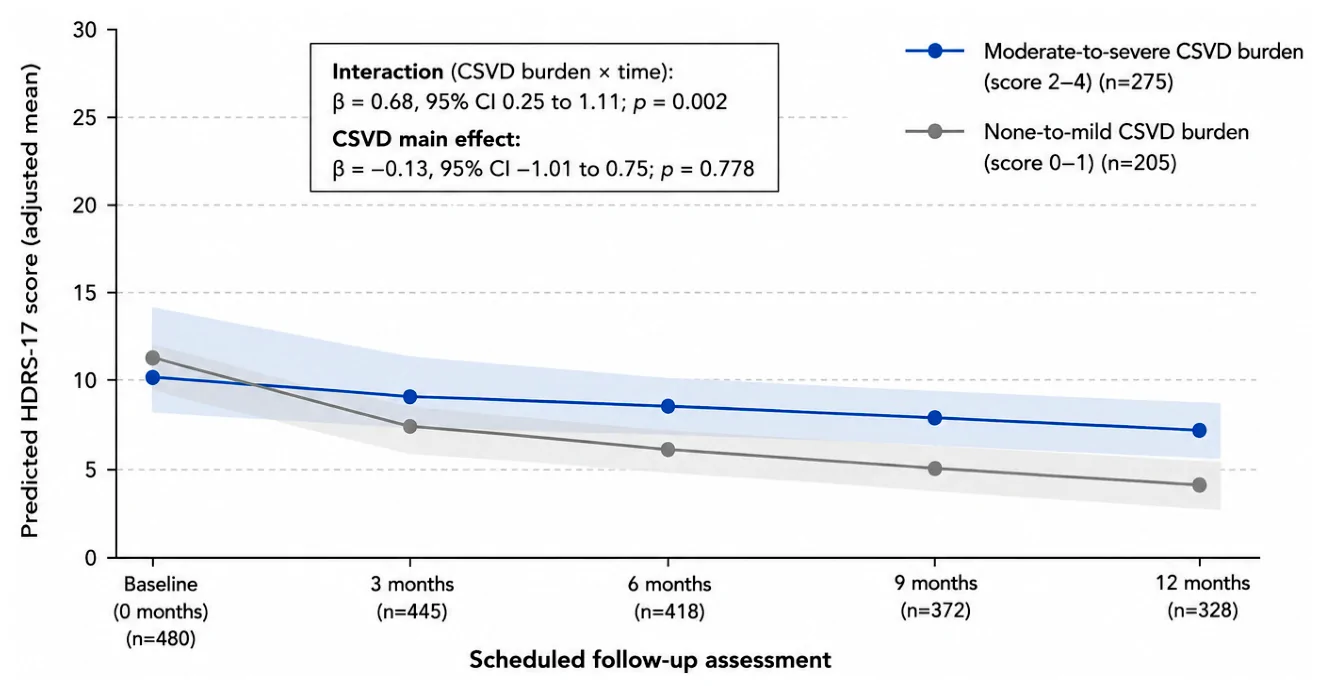
**Adjusted longitudinal trajectories of depressive symptom severity according to baseline MRI-defined cerebral small vessel disease burden.** Footnotes: Linear mixed-effects models incorporated participant-specific random intercepts and included all available repeated HDRS-17 measurements. Moderate-to-severe CSVD burden was defined as a Global CSVD Score of 2–4, with zero-to-mild CSVD burden (score 0–1) as the reference category. Follow-up time was entered as an ordinal numeric variable representing scheduled visit sequence (baseline = 0, 3 months = 1, 6 months = 2, and 9–12 months = 3). The CSVD burden × follow-up time interaction indicates that longitudinal HDRS-17 trajectories differed between CSVD burden groups; the direction of this difference is illustrated by the model-predicted trajectories in Figure 3. Abbreviations: CSVD, cerebral small vessel disease; HDRS-17, 17-item Hamilton Depression Rating Scale; MRI, magnetic resonance imaging.

Figure 3 illustrates the adjusted longitudinal trajectories of depressive symptom severity according to baseline MRI-defined CSVD burden. The trajectories differed between CSVD burden groups, consistent with the significant CSVD burden × follow-up time interaction in the linear mixed-effects model (Table 2), with a less favorable longitudinal depressive symptom trajectory among participants with moderate-to-severe CSVD burden.

### 3.3. New-Onset Post-Stroke Depression

Among the 427 participants included in the complete-case analysis, moderate-to-severe baseline MRI-defined CSVD burden was not independently associated with incident post-stroke depression after multivariable adjustment (Table 3). The association remained nonsignificant across sequential adjustment models, including the fully adjusted model (aOR 1.27, 95% CI [0.77–2.12]; *p* = 0.351).

Exploratory analyses identified a nominal interaction between baseline MRI-defined CSVD burden and admission NIHSS score in the fully adjusted model (aOR: 0.83; 95% CI [0.70–1.00]; *p* = 0.048) (Supplementary Table S2). This exploratory interaction was interpreted cautiously as hypothesis-generating rather than confirmatory. Among the individual MRI-defined CSVD markers, moderate-to-severe white matter hyperintensities showed the strongest nominal association with incident PSD (aOR: 2.10; 95% CI [1.15–3.84]; *p* = 0.016) (Supplementary Table S3); however, this finding did not meet the Bonferroni-adjusted significance threshold of *p* < 0.0125. Lacunes, cerebral microbleeds, and enlarged perivascular spaces were not independently associated with incident PSD.

### 3.4. Persistent Post-Stroke Depression

Moderate-to-severe baseline MRI-defined CSVD burden was not independently associated with persistent post-stroke depression after multivariable adjustment (aOR 1.11, 95% CI [0.62–1.99]; *p* = 0.734) (Table 4).

### 3.5. Functional Outcomes

After multivariable adjustment for demographic, vascular, and stroke-related covariates, moderate-to-severe baseline MRI-defined CSVD burden was independently associated with poor functional outcome at 90 days (mRS > 2) (aOR 1.56, 95% CI [1.04–2.32]; *p* = 0.030) (Table 4). Baseline anxiety symptoms were also independently associated with poor functional outcome at 90 days (aOR 1.58, 95% CI [1.01–2.47]; *p* = 0.047). Persistent post-stroke depression was associated with higher odds of poor functional outcome at 90 days; however, this association did not reach statistical significance (aOR 1.88, 95% CI [0.98–3.61]; *p* = 0.056).

### 3.6. Cognitive Outcome

Among participants with MRI-defined CSVD and available 6-month MoCA data, 345 were included in the cognitive analysis, comprising 143 with zero-to-mild CSVD burden and 202 with moderate-to-severe CSVD burden. Mean MoCA scores were lower in the moderate-to-severe CSVD group both at baseline (18.80 ± 3.57 vs. 21.38 ± 3.96) and at 6 months (19.05 ± 3.94 vs. 21.80 ± 4.20; *p* < 0.001). Cognitive impairment at 6 months, defined as MoCA < 26, was present in 192/202 (95.0%) participants with moderate-to-severe CSVD compared with 116/143 (81.1%) participants with zero-to-mild CSVD (*p* < 0.001). However, mean change in MoCA from baseline to 6 months did not differ significantly between groups (+0.25 vs. +0.42 points; *p* = 0.391).

After multivariable adjustment, moderate-to-severe baseline MRI-defined CSVD burden was associated with higher odds of cognitive impairment at 6 months, although the association did not reach statistical significance (aOR 1.50, 95% CI [0.97–2.31]; *p* = 0.069) (Table 4). In contrast, baseline anxiety symptoms were independently associated with higher odds of cognitive impairment at 6 months (aOR 6.65, 95% CI [3.91–11.29]; *p* < 0.001).

**Figure 4:**
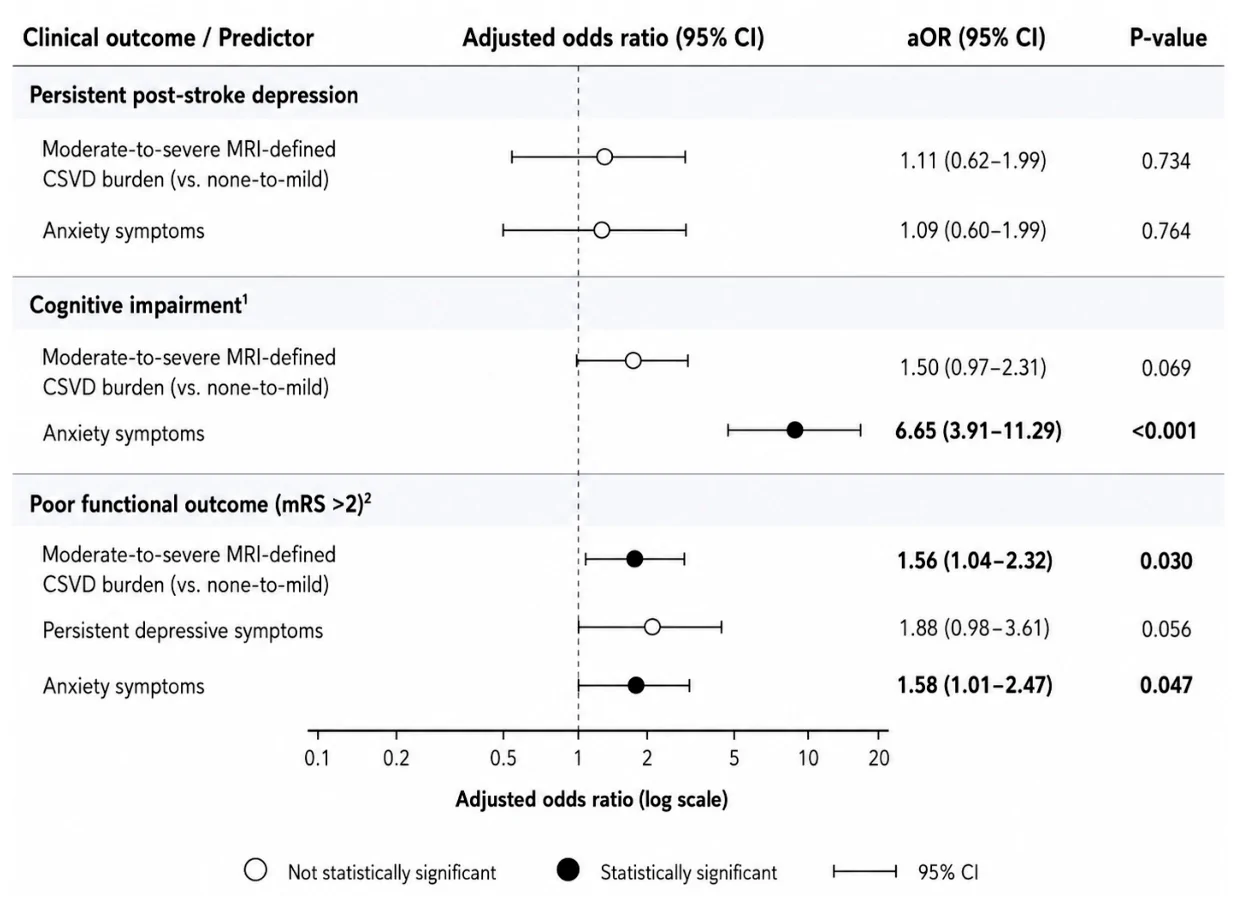
**Adjusted associations between baseline MRI-defined cerebral small vessel disease (CSVD) burden and secondary post-stroke clinical outcomes.** Forest plot showing adjusted odds ratios (aORs) and 95% confidence intervals (CIs) derived from separate multivariable logistic regression models. Models were adjusted for prespecified demographic and clinical covariates as described in the Statistical Analysis subsection. Moderate-to-severe MRI-defined CSVD burden was defined as a Global CSVD Score of 2–4, with zero-to-mild CSVD burden (score 0–1) as the reference category. Cognitive impairment ^1^ was defined as a Montreal Cognitive Assessment (MoCA) score <26 at the 6-month follow-up assessment. Poor functional outcome ^2^ was defined as a modified Rankin Scale (mRS) score > 2 at the prespecified follow-up assessment. The vertical dashed line indicates the null effect (aOR = 1.0). Filled circles indicate statistically significant associations (*p* < 0.05), whereas open circles indicate associations that did not reach statistical significance; horizontal lines represent 95% CIs. Abbreviations: aOR, adjusted odds ratio; CI, confidence interval; CSVD, cerebral small vessel disease; MRI, magnetic resonance imaging; MoCA, Montreal Cognitive Assessment; mRS, modified Rankin Scale.

Figure 4 summarizes the adjusted associations between baseline MRI-defined CSVD burden and secondary post-stroke clinical outcomes. Moderate-to-severe CSVD burden was independently associated with poor functional outcome at 90 days, whereas its associations with persistent PSD and cognitive impairment at 6 months were not statistically significant after multivariable adjustment.

## 4. Discussion

In this prospective longitudinal cohort, greater MRI-defined CSVD burden was most consistently associated with the longitudinal trajectory of depressive symptom severity and with functional outcome after stroke. Participants with moderate-to-severe CSVD burden demonstrated a less favorable depressive symptom trajectory over follow-up, reflected by a significant CSVD burden × follow-up time interaction, while greater CSVD burden was also independently associated with poorer functional recovery. These findings are consistent with previous studies linking greater total CSVD burden to adverse functional outcomes after stroke [55,56,57,58,59,60]. In contrast, baseline CSVD burden was not independently associated with incident or persistent PSD, or with global cognitive impairment after multivariable adjustment. Taken together, these findings suggest that the clinical relevance of CSVD in post-stroke recovery may be more apparent in longitudinal symptom trajectories and functional outcomes than in dichotomized neuropsychiatric outcomes assessed at individual time points.

These findings extend previous research on post-stroke neuropsychiatric complications, including emerging evidence from Saudi Arabia [61,62], and support further evaluation of MRI-defined CSVD burden as a complementary marker of longer-term emotional and functional recovery after stroke [63]. Although prevalence estimates vary according to study design, patient characteristics, assessment instruments, and timing of evaluation, regional studies consistently identify post-stroke depression as an important component of stroke recovery [61,62,64]. This is consistent with international evidence linking post-stroke depression to poorer quality of life, greater disability, impaired rehabilitation, and less favorable long-term functional outcomes [65,66,67,68].

Participants with moderate-to-severe CSVD burden had a greater vascular risk burden at baseline, consistent with the established contribution of hypertension, diabetes mellitus, dyslipidemia, smoking, and other vascular risk factors to the development and progression of cerebral small vessel disease. Nevertheless, baseline stroke severity, stroke subtype, lesion characteristics, depressive symptom severity, and cognitive performance were otherwise broadly similar between groups. Therefore, the observed follow-up differences may be explained by conventional vascular risk factors or characteristics of the index stroke, although residual confounding cannot be excluded. Further, MRI-defined CSVD burden remained associated with longitudinal depressive symptom trajectory and functional outcome after adjustment for demographic, vascular, and stroke-related factors. These findings suggest that greater MRI-defined CSVD burden is associated with differences in the course of depressive symptoms after stroke and raise the possibility that cumulative microvascular brain injury may contribute to neuropsychiatric recovery patterns [20,55,59,60,69].

A key finding of the present study was the significant difference in longitudinal depressive symptom trajectories according to baseline MRI-defined CSVD burden. Participants with moderate-to-severe CSVD burden demonstrated a less favorable depressive symptom trajectory over follow-up, as reflected by the significant CSVD burden × follow-up time interaction in the linear mixed-effects model (Figure 3). These findings suggest that greater MRI-defined CSVD burden is associated with differences in the course of depressive symptoms after stroke, raising the possibility that cumulative microvascular brain injury may contribute to patterns of neuropsychiatric recovery. Previous studies have shown that post-stroke depressive symptoms may follow heterogeneous longitudinal courses [5,7,70,71,72], while greater MRI-defined CSVD burden has also been associated with post-stroke depressive symptoms [21,24,25]. Our findings extend this literature by demonstrating an association between baseline cumulative CSVD burden and subsequent depressive symptom trajectory. Although the trajectory difference was statistically significant, the present study did not establish a minimum clinically important difference for the HDRS-17 in post-stroke patients. Therefore, the clinical magnitude of this difference should be interpreted cautiously and distinguished from statistical significance.

Despite the observed difference in longitudinal depressive symptom trajectories, baseline MRI-defined CSVD burden was not independently associated with either incident or persistent PSD after multivariable adjustment. This apparent discrepancy likely reflects differences between dichotomized outcomes assessed at discrete time points and longitudinal analyses incorporating repeated symptom measurements rather than genuinely contradictory findings. Whereas incident and persistent post-stroke depression classify participants according to whether a predefined diagnostic threshold is reached, linear mixed-effects models capture changes in depressive symptom severity across repeated assessments. Accordingly, CSVD burden may be more closely associated with the longitudinal evolution of depressive symptom severity than with dichotomized depression status at individual follow-up assessments [73,74,75,76,77]. This interpretation is consistent with the American Heart Association/American Stroke Association scientific statement emphasizing the importance of ongoing recognition and management of post-stroke depression throughout recovery [6].

Beyond depressive symptoms, anxiety emerged as one of the strongest correlates of post-stroke cognitive impairment in our cohort, with more than sixfold higher odds of cognitive impairment after multivariable adjustment (aOR 6.65, 95% CI [3.91–11.29]). Although the direction of this association cannot be determined from the present study, it is consistent with evidence that emotional and cognitive disturbances frequently coexist after stroke and may interact during recovery [76,78,79]. Depressive symptoms have also been linked to cognitive performance and cognitive reserve in other populations, further supporting the close relationship between emotional and cognitive health [80]. This relationship may be particularly relevant in CSVD, which is increasingly conceptualized as a diffuse disorder affecting distributed brain networks rather than producing only isolated focal deficits. Neuroimaging studies have linked anxiety and depressive symptoms after subcortical stroke with MRI-defined brain injury [81], while recent work in CSVD has shown that disrupted brain structure–function coupling is associated with cognitive dysfunction [82]. Together, these findings suggest an interrelationship between emotional symptoms, cognitive impairment, and underlying structural brain abnormalities after stroke. However, the present observational data cannot determine whether these associations are directly attributable to CSVD-related network dysfunction or instead reflect shared vascular, inflammatory, psychological, or other clinical factors [83,84,85,86,87,88,89,90,91].

The exploratory analysis suggested that the association between CSVD burden and PSD symptoms may vary according to baseline stroke severity. Although the interaction between CSVD burden and baseline NIHSS score yielded a nominal *p*-value of 0.048 in the fully adjusted model, this analysis was exploratory; therefore, it should be interpreted cautiously as hypothesis-generating rather than confirmatory. The relative influence of pre-existing microvascular brain injury may be more apparent in patients with milder strokes, in whom longer-term emotional recovery is less dominated by the severity of the acute neurological insult. This interpretation is consistent with evidence that depressive and anxiety symptoms remain clinically relevant after minor stroke or transient ischemic attack despite relatively favorable neurological outcomes [92]. Conversely, the observed interaction in our cohort raises the possibility that, among patients with more severe strokes, the effects of acute neurological injury and stroke-related disability may reduce the relative contribution of underlying CSVD to emotional recovery. Given the exploratory nature of this analysis, this interpretation remains speculative and requires confirmation in larger prospective cohorts.

These findings may have implications for individualized post-stroke follow-up and rehabilitation. Greater MRI-defined CSVD burden may help identify patients who warrant closer monitoring of emotional and functional recovery; however, its incremental prognostic value beyond established clinical predictors requires further validation. Contemporary stroke rehabilitation increasingly emphasizes assessment and treatment tailored to the specific neurological and functional deficits of individual patients. Recent randomized rehabilitation research, including the study by Canli et al. [93], illustrates the ongoing evaluation of targeted interventions for specific post-stroke impairments. Although the intervention examined in that trial was not evaluated in the present study, such work supports the broader principle of individualized rehabilitation planning. Therefore, our findings support further evaluation of CSVD burden as part of a broader clinical assessment rather than as a basis for any specific therapeutic intervention. Future studies should determine whether incorporating CSVD burden into validated prognostic models improves risk stratification and informs post-stroke follow-up and clinical decision-making.

The present study has several strengths. Its prospective longitudinal design enabled assessment of recovery trajectories across multiple follow-up visits rather than reliance on isolated cross-sectional measurements. Repeated assessments across follow-up visits allowed emotional, cognitive, neurological, and functional recovery to be characterized longitudinally within the same cohort. Importantly, neuropsychiatric and cognitive assessments were conducted face to face rather than by telephone. PSD was identified through clinical diagnostic assessment based on DSM-5 criteria [44], while the HDRS-17 was used to quantify depressive symptom severity longitudinally [33,34,35]. Anxiety and cognitive function were also assessed directly using the GAD-7 [31,32] and MoCA [36,38], respectively, by a neurology resident during follow-up visits. This approach reduced reliance on remote or self-administered assessment and allowed both categorical neuropsychiatric outcomes and longitudinal changes in symptom severity to be evaluated.

The imaging methodology provides additional strengths. CSVD burden was characterized using a STRIVE-based global CSVD score, providing a standardized assessment of cumulative MRI-defined small vessel disease. All MRI examinations included in the CSVD analyses were acquired at 1.5 T, providing consistency in imaging field strength across participants and minimizing variability related to field strength. MRI markers were independently assessed by two readers, including a neurology resident and a consultant neuroradiologist, with disagreements resolved by consensus and final adjudication by the consultant neuroradiologist. By integrating standardized MRI-defined CSVD burden with repeated face-to-face neuropsychiatric, cognitive, and functional assessments, the study provides a multidimensional longitudinal characterization of post-stroke recovery. To our knowledge, relatively few studies have simultaneously examined these domains over time using a global MRI-defined CSVD burden score. The inclusion of patients with predominantly mild-to-moderate stroke is also clinically relevant, as substantial emotional and cognitive morbidity may persist despite comparatively favorable neurological recovery [78,92].

Several limitations should be acknowledged. First, the single-center observational design precludes causal inference and may limit generalizability, particularly beyond the study population and healthcare setting in Saudi Arabia. Hence, validation in geographically and ethnically diverse multi-center cohorts is required. Second, CSVD burden could only be assessed in participants who underwent brain MRI, introducing potential selection bias because MRI acquisition was determined by routine clinical practice rather than a standardized research protocol. However, baseline patient characteristics were broadly comparable between participants who underwent MRI and those who did not, with only small imbalances for most variables (Supplementary Table S1). Third, although MRI-defined CSVD markers were independently evaluated by two blinded assessors with consensus adjudication of discrepancies, formal inter-rater reliability coefficients were not prospectively collected. Fourth, CSVD burden was quantified using a validated STRIVE-based global score rather than advanced quantitative neuroimaging techniques. Fifth, premorbid cognitive status and psychiatric history were not systematically assessed using validated informant-based instruments.

Sixth, although antidepressant initiation was incorporated as a time-varying covariate in the sensitivity analysis, treatment was clinically indicated rather than randomly assigned. Therefore, residual confounding by indication and by antidepressant class, dose, adherence, switching, and discontinuation cannot be excluded. Moreover, the treatment variable represented documented antidepressant initiation before each assessment rather than confirmed continuous exposure throughout follow-up. Although rehabilitation and physical therapy were provided according to clinical need as part of routine post-stroke care, detailed information regarding the frequency, duration, and intensity of rehabilitation sessions was not systematically available. Therefore, differences in rehabilitation exposure, psychosocial support, and other post-stroke interventions may have contributed to the observed outcomes. Finally, although the 12-month follow-up period allowed assessment of cognitive outcomes during the first year after stroke, it was insufficient to characterize longer-term cognitive decline or incident vascular dementia.

Despite these limitations, greater MRI-defined CSVD burden remained associated with differences in longitudinal depressive symptom trajectories and poorer functional outcome after multivariable adjustment. The association with depressive symptom trajectories also remained statistically significant after incorporating antidepressant initiation as a time-varying covariate, supporting the robustness of the main longitudinal findings. These results support further evaluation of CSVD burden as a complementary imaging marker of post-stroke emotional and functional recovery. Future multi-center studies incorporating quantitative neuroimaging, detailed time-varying treatment and rehabilitation data, and longer follow-up are needed to validate these associations and determine whether CSVD burden provides clinically meaningful incremental information beyond established clinical predictors across diverse stroke populations.

## 5. Conclusions

Greater MRI-defined CSVD burden was associated with a less favorable longitudinal depressive symptom trajectory and poorer functional recovery after stroke. In contrast, baseline CSVD burden was not independently associated with incident or persistent PSD or global cognitive impairment after multivariable adjustment. These findings suggest that cumulative MRI-defined CSVD burden may provide complementary information regarding longitudinal emotional and functional recovery alongside conventional measures of acute stroke severity; however, its incremental prognostic value and clinical utility require further validation. Future multi-center studies incorporating quantitative neuroimaging, longer-term follow-up, and diverse stroke populations are needed to confirm these associations and determine whether CSVD assessment improves risk stratification and informs individualized post-stroke monitoring and rehabilitation.

## Figures and Tables

**Table 1:** Baseline demographic, clinical, vascular, and stroke characteristics according to baseline cerebral small vessel disease (CSVD) burden among patients undergoing brain MRI (n = 480).

Study Variable	Zero-to-Mild CSVD Burden (Score 0–1), n = 205	Moderate-to-Severe CSVD Burden (Score 2–4), n = 275	Test Statistic (*t*, *U*, χ^2^, or Fisher’s Exact, as Appropriate)	*p* Value
Demographic characteristics				
Age, years, mean ± SD	54.1 ± 14.3	63.8 ± 13.0	−7.613	<0.001
Sex			0.013	0.911
Female, n (%)	92 (44.9)	126 (45.8)		
Male, n (%)	113 (55.1)	149 (54.2)		
Educational attainment ≥ 12 years			0.033	0.856
No, n (%)	130 (63.4)	171 (62.2)		
Yes, n (%)	75 (36.6)	104 (37.8)		
Socioeconomic status			3.841	0.147
Excellent, n (%)	50 (24.4)	48 (17.5)		
Good, n (%)	115 (56.1)	162 (58.9)		
Poor, n (%)	40 (19.5)	65 (23.6)		
Clinical characteristics				
Pre-existing depression			0.007	0.933
No, n (%)	184 (89.8)	245 (89.1)		
Yes, n (%)	21 (10.2)	30 (10.9)		
Baseline NIHSS score, median (range)	8 (0–24)	7 (0–26)	30213.0	0.177
Vascular risk factors				
Hypertension, n (%)	109 (53.2)	223 (81.1)	41.631	<0.001
Diabetes mellitus, n (%)	66 (32.2)	111 (40.4)	3.025	0.082
Dyslipidemia, n (%)	100 (48.8)	121 (44.0)	0.897	0.344
Current smoking, n (%)	44 (21.5)	52 (18.9)	0.333	0.564
Atrial fibrillation, n (%)	12 (5.9)	25 (9.1)	1.305	0.253
Prior stroke/TIA, n (%)	28 (13.7)	40 (14.5)	0.021	0.886
Vascular risk burden category			8.027	0.005
Low (0–1 risk factors), n (%)	86 (42.0)	80 (29.1)		
Moderate-to-high (≥2 risk factors), n (%)	119 (58.0)	195 (70.9)		
Stroke characteristics				
Stroke type			0.541	0.462
Ischemic stroke, n (%)	172 (83.9)	221 (80.4)		
Intracerebral hemorrhage, n (%)	33 (16.1)	54 (19.6)		
Ischemic stroke subtype			3.684	0.450
Large-artery atherosclerosis, n (%)	38 (22.1)	57 (25.8)		
Small-vessel disease, n (%)	46 (26.7)	47 (21.3)		
Cardioembolic stroke, n (%)	35 (20.3)	40 (18.1)		
ESUS, n (%)	36 (20.9)	45 (20.4)		
Other determined causes, n (%)	17 (9.9)	32 (14.5)		
Deep lesion involvement, n (%)	72 (35.1)	91 (33.1)	0.135	0.713

Footnotes: Of the 550 enrolled patients with acute stroke, 480 underwent brain MRI and were included in the MRI-defined CSVD analyses. Data are presented as mean ± standard deviation, median (range), or n (%), as appropriate. Continuous variables were compared using the independent-samples *t* test or Mann–Whitney U test, as appropriate, whereas categorical variables were compared using the chi-square test or Fisher’s exact test. Vascular risk burden was calculated from hypertension, diabetes mellitus, dyslipidemia, current smoking, atrial fibrillation, and prior stroke/TIA. Ischemic stroke subtype was evaluated only among participants with ischemic stroke; participants with intracerebral hemorrhage were excluded from the ischemic stroke subtype analysis. Abbreviations: CSVD, cerebral small vessel disease; ESUS, embolic stroke of undetermined source; ICH, intracerebral hemorrhage; MRI, magnetic resonance imaging; NIHSS, National Institutes of Health Stroke Scale; SD, standard deviation; TIA, transient ischemic attack.

**Table 2:** Linear mixed-effects model of longitudinal depressive symptom severity according to baseline MRI-defined CSVD burden.

Model Term	β Coefficient	95% CI	*p* Value
Follow-up time	1.38	1.06–1.70	<0.001
Moderate-to-severe CSVD burden	−0.13	−1.01–0.75	0.778
CSVD burden × follow-up time	0.68	0.25–1.11	0.002
Age	0.01	−0.01–0.04	0.209
Baseline NIHSS score	0.23	0.17–0.29	<0.001
Baseline HDRS-17 score	0.42	0.32–0.51	<0.001

Footnotes: Linear mixed-effects models included 480 participants contributing 1625 HDRS-17 observations, incorporated participant-specific random intercepts, and included all available repeated HDRS-17 measurements. Moderate-to-severe CSVD burden was defined as a Global CSVD Score of 2–4, with zero-to-mild CSVD burden (score 0–1) as the reference category. Follow-up time was entered as an ordinal numeric variable representing scheduled visit sequence (baseline = 0, 3 months = 1, 6 months = 2, and 9–12 months = 3). A positive CSVD burden × follow-up time coefficient indicates a less favorable longitudinal depressive symptom trajectory among participants with moderate-to-severe CSVD burden. Abbreviations: CI, confidence interval; CSVD, cerebral small vessel disease; HDRS-17, 17-item Hamilton Depression Rating Scale; NIHSS, National Institutes of Health Stroke Scale.

**Table 3:** Multivariable logistic regression analysis of the association between baseline cerebral small vessel disease (CSVD) burden and incident post-stroke depression among participants without pre-existing depression and with complete analytical data (n = 427).

Predictor	Model 1 aOR (95% CI)	*p* Value	Model 2 aOR (95% CI)	*p* Value	Model 3 aOR (95% CI)	*p* Value
Zero-to-mild CSVD burden (score 0–1)	Reference	—	Reference	—	Reference	—
Moderate-to-severe CSVD burden (score 2–4)	1.32 (0.79–2.19)	0.292	1.42 (0.84–2.42)	0.196	1.27 (0.77–2.12)	0.351

Footnotes: Analyses were restricted to participants without documented pre-existing depression who underwent brain MRI. Two participants with missing outcome or covariate data were excluded from the complete-case multivariable analysis, resulting in a final analytical sample of 427 participants. Incident post-stroke depression was defined as clinically significant depressive symptoms (HDRS-17 ≥ 10) occurring during follow-up among participants without pre-existing depression. Model 1 was adjusted for age and sex. Model 2 was additionally adjusted for baseline NIHSS score and a quadratic NIHSS term (NIHSS^2^) to account for potential nonlinear effects of stroke severity; the quadratic term was included in model estimation but is not displayed. Model 3 was additionally adjusted for educational attainment, vascular risk burden category, stroke type, and lesion location. Moderate-to-severe CSVD burden was defined as a Global CSVD Score of 2–4, with zero-to-mild CSVD burden defined as a score of 0–1. Odds ratios > 1.0 indicate higher odds of incident post-stroke depression. A two-sided *p* < 0.05 was considered statistically significant. Abbreviations: aOR, adjusted odds ratio; CI, confidence interval; CSVD, cerebral small vessel disease; HDRS-17, 17-item Hamilton Depression Rating Scale; MRI, magnetic resonance imaging; NIHSS, National Institutes of Health Stroke Scale; PSD, post-stroke depression.

**Table 4:** Multivariable associations between baseline cerebral small vessel disease burden and secondary clinical outcomes after stroke.

Outcome	Predictor	aOR (95% CI)	*p* Value
Persistent post-stroke depression	Moderate-to-severe CSVD burden	1.11 (0.62–1.99)	0.734
	Baseline anxiety symptoms	1.09 (0.60–1.99)	0.764
Cognitive impairment at 6 months (MoCA < 26)	Moderate-to-severe CSVD burden	1.50 (0.97–2.31)	0.069
	Baseline anxiety symptoms	6.65 (3.91–11.29)	<0.001
Poor functional outcome (mRS > 2)	Moderate-to-severe CSVD burden	1.56 (1.04–2.32)	0.030
	Persistent depressive symptoms	1.88 (0.98–3.61)	0.056
	Baseline anxiety symptoms	1.58 (1.01–2.47)	0.047

Footnotes: Estimates are presented as adjusted odds ratios (aORs) with 95% confidence intervals derived from separate multivariable logistic regression models for each outcome. Moderate-to-severe CSVD burden was defined as a Global CSVD Score of 2–4, with zero-to-mild CSVD burden (score 0–1) as the reference category. Persistent post-stroke depression was defined as clinically significant depressive symptoms (HDRS-17 ≥ 10) present at two or more follow-up assessments. Cognitive impairment was defined as a Montreal Cognitive Assessment (MoCA) score < 26 at the 6-month follow-up assessment. Poor functional outcome was defined as a modified Rankin Scale (mRS) score > 2 at the 90-day follow-up assessment. Models were adjusted for prespecified demographic and clinical covariates, including age, sex, educational attainment, baseline NIHSS score, vascular risk burden, stroke type, and lesion location, with additional outcome-specific covariates included as specified in the Statistical Analysis subsection. A two-sided *p* value < 0.05 was considered statistically significant. Abbreviations: aOR, adjusted odds ratio; CI, confidence interval; CSVD, cerebral small vessel disease; HDRS-17, 17-item Hamilton Depression Rating Scale; MoCA, Montreal Cognitive Assessment; mRS, modified Rankin Scale.

## Data Availability

The datasets presented in this article are not publicly available because they contain potentially identifiable participant information and are subject to institutional ethical restrictions. Requests to access the datasets should be directed to the corresponding author. De-identified data may be made available upon reasonable request, subject to approval by the Institutional Review Board.

## Supplementary Materials

The following supporting information can be downloaded at https://www.mdpi.com/article/10.3390/neurolint18090179/s1, Table S1: Baseline characteristics of participants who underwent brain MRI versus those who did not; Table S2: Exploratory interaction analysis between baseline MRI-defined cerebral small vessel disease (CSVD) burden and baseline stroke severity for incident post-stroke depression among participants without documented pre-existing depression and with complete analytical data (n = 427); Table S3: Association between individual MRI-defined cerebral small vessel disease markers and incident post-stroke depression among participants without documented pre-existing depression and with complete analytical data (n = 427); Table S4: Sensitivity analysis of longitudinal depressive symptom trajectories after adjustment for antidepressant initiation before assessment.

## Abbreviations

The following abbreviations are used in this manuscript:

PSD	post-stroke depression
CSVD	cerebral small vessel disease
AIS	acute ischemic stroke
TIA	transient ischemic attack
ICH	intracerebral hemorrhage
CVST	cerebral venous sinus thrombosis
STROBE	Strengthening the Reporting of Observational Studies in Epidemiology
STRIVE	Standards for Reporting Vascular Changes on Neuroimaging
HDRS-17	17-item Hamilton Depression Rating Scale
GAD-7	Generalized Anxiety Disorder-7
MoCA	Montreal Cognitive Assessment
NIHSS	National Institutes of Health Stroke Scale
mRS	modified Rankin Scale
DSM-5	Diagnostic and Statistical Manual of Mental Disorders, Fifth Edition
TOAST	Trial of Org 10172 in Acute Stroke Treatment
SD	standard deviation
IQR	interquartile range
VIFs	variance inflation factors
AUC	area under the receiver operating characteristic curve
aORs	adjusted odds ratios
CIs	confidence intervals
MRI	magnetic resonance imaging
