# Supplementary material for: Magnetic Resonance Imaging-Defined Cerebral Small Vessel Disease Burden and Longitudinal Neuropsychiatric and Functional Recovery After Stroke: A Prospective Cohort Study
Source: Neurol Int. 2026 Sep 21;18(9):179. doi: 10.3390/neurolint18090179 (PMC13610538; doi:10.3390/neurolint18090179)
Supplement: Supplementary file 1 [file neurolint-18-00179-s001.zip › neurolint-4519473-supplementary.pdf]

Supplementary Materials: Magnetic Resonance Imaging-defined Cerebral Small Vessel Disease Burden and Longitudinal Neuropsychiatric and Functional Recovery After Stroke: A Prospective Cohort Study

Aishah Ibrahim Albakr [1,*], Alia Alokley [2], Feras Alsulaiman [1], Abdulrahman Abdulwahab [3], Mohammad AlWatban [3], Dalal Albakr [4], Feras A. Al-Awad [4], Mustafa Ahmed Alqarni [1], Saud A. Alnaaim [2], Ali Hafiz Alhashim [1], Noman Ishaque [5], Modhi AlKhaldi [1], Azra Zafar [1], Fatimah AlSaihati [1], Saleh Saad Alotaibi [1], Firas Saad Alahmari [1] and Norah A. AlKhaldi [1]

Supplementary Table S1. Baseline characteristics of participants who underwent brain MRI versus those who did not.

Characteristic	MRI performed (n = 480)	MRI not performed (n = 70)	SMD	*p*-value
Age, years, mean ± SD	59.7 ± 14.4	60.1 ± 17.1	0.03	0.560
Male sex, n (%)	262 (54.6)	41 (58.6)	0.08	0.531
Education ≥12 years, n (%)	179 (37.3)	32 (45.7)	0.17	0.176
Pre-existing depression, n (%)	51 (10.6)	5 (7.1)	0.12	0.368
Baseline NIHSS score, mean ± SD	7.6 ± 5.3	8.7 ± 5.2	0.21	0.071
Hypertension, n (%)	332 (69.2)	48 (68.6)	0.01	0.920
Diabetes mellitus, n (%)	177 (36.9)	25 (35.7)	0.02	0.851
Dyslipidemia, n (%)	221 (46.0)	29 (41.4)	0.09	0.469
Current smoking, n (%)	96 (20.0)	15 (21.4)	0.04	0.781
Atrial fibrillation, n (%)	37 (7.7)	7 (10.0)	0.08	0.509
Prior stroke/TIA, n (%)	68 (14.2)	9 (12.9)	0.04	0.768

Footnote: SMDs were used to assess baseline imbalance between participants who underwent MRI and those who did not. An absolute SMD <0.20 was considered small. Baseline characteristics were generally well balanced between groups, although baseline NIHSS showed a modest imbalance (SMD = 0.21).
Abbreviations: MRI, magnetic resonance imaging; NIHSS, National Institutes of Health Stroke Scale; SD, standard deviation; SMD, standardized mean difference; TIA, transient ischemic attack.

Supplementary Table S2. Exploratory interaction analysis between baseline MRI-defined cerebral small vessel disease (CSVD) burden and baseline stroke severity for incident post-stroke depression among participants without documented pre-existing depression and with complete analytical data (n = 427).

Model / Predictor	aOR	95% CI	*p*-value
Model 1a: Demographic-adjusted model			
Moderate-to-severe CSVD burden	0.91	0.25–3.38	0.892

CSVD burden × baseline NIHSS interaction	0.85	0.71–1.00	0.053
Model 1b: Stroke severity-adjusted model			
Moderate-to-severe CSVD burden	0.43	0.05–3.43	0.424
CSVD burden × baseline NIHSS interaction	2.08	0.80–5.37	0.132
Model 1c: Fully adjusted model			
Moderate-to-severe CSVD burden	0.56	0.13–2.51	0.450
CSVD burden × baseline NIHSS interaction	0.83	0.70–1.00	0.048

Footnotes: Analyses were restricted to participants without documented pre-existing depression who underwent brain MRI and had complete analytical data (n = 427). Incident post-stroke depression was defined as clinically significant depressive symptoms (HDRS-17 ≥10) occurring during follow-up. Moderate-to-severe CSVD burden was defined as a Global CSVD Score of 2–4, with none-to-mild CSVD burden (score 0–1) as the reference category. Model 1a was adjusted for age and sex. Model 1b was additionally adjusted for baseline NIHSS score and a quadratic NIHSS term (NIHSS^2) to account for potential nonlinear effects of stroke severity. Model 1c was additionally adjusted for educational attainment, vascular risk burden category, stroke type, and lesion location. Interaction analyses were exploratory. The CSVD burden × baseline NIHSS interaction reached nominal statistical significance in the fully adjusted model (p = 0.048) and was therefore interpreted cautiously as hypothesis-generating rather than confirmatory. Bonferroni correction for the four individual CSVD-marker analyses was performed separately, as described in Supplementary Table S3.
Abbreviations: aOR, adjusted odds ratio; CI, confidence interval; CSVD, cerebral small vessel disease; HDRS-17, 17-item Hamilton Depression Rating Scale; MRI, magnetic resonance imaging; NIHSS, National Institutes of Health Stroke Scale.

Supplementary Table S3. Association between individual MRI-defined cerebral small vessel disease markers and incident post-stroke depression among participants without documented pre-existing depression and with complete analytical data (n = 427).

**CSVD Marker**	**aOR**	**95% CI**	***p-value***	**Bonferroni-adjusted *p*-value**
White matter hyperintensities (moderate-to-severe vs absent-to-mild)	2.10	1.15–3.84	0.016	0.064
Lacunes (present vs absent)	1.95	0.92–4.12	0.081	0.324
Cerebral microbleeds (present vs absent)	1.54	0.78–3.05	0.214	0.856
Enlarged perivascular spaces (moderate-to-severe vs absent-to-mild)	1.22	0.65–2.28	0.538	1.000

Footnotes: Analyses were restricted to participants without documented pre-existing depression and with complete analytical data (n = 427). Incident post-stroke depression was defined as clinically significant depressive symptoms (HDRS-17 ≥10) occurring during follow-up. White matter hyperintensities and enlarged perivascular spaces were dichotomized as none-to-mild or moderate-to-severe according to established visual rating scales. All four CSVD markers were entered simultaneously into the multivariable logistic regression model; therefore, each adjusted odds ratio represents the independent association of the corresponding marker after adjustment for the remaining CSVD markers and prespecified covariates, including age, sex, educational attainment, baseline NIHSS score, and vascular risk burden category. Bonferroni correction was applied for four marker comparisons, yielding a corrected significance threshold of ***p-value*** < 0.0125.

Abbreviations: aOR, adjusted odds ratio; CI, confidence interval; CSVD, cerebral small vessel disease; MRI, magnetic resonance imaging.

Supplementary Table S4. Sensitivity analysis of longitudinal depressive symptom trajectories after adjustment for antidepressant initiation before assessment.

Model term	Primary model, β (95% CI)	*p-value*	Antidepressant-adjusted model, β (95% CI)	*p-value*
Follow-up time	1.38 (1.06 to 1.70)	<0.001	1.12 (0.79 to 1.44)	<0.001
Moderate-to-severe CSVD burden	−0.13 (−1.01 to 0.75)	0.778	−0.14 (−0.99 to 0.72)	0.756
CSVD burden × follow-up time	0.68 (0.25 to 1.11)	0.002	0.60 (0.17 to 1.02)	0.006
Age	0.01 (−0.01 to 0.04)	0.209	0.01 (−0.01 to 0.03)	0.326
Baseline NIHSS score	0.23 (0.17 to 0.29)	<0.001	0.21 (0.15 to 0.26)	<0.001
Baseline HDRS-17 score	0.42 (0.32 to 0.51)	<0.001	0.40 (0.31 to 0.49)	<0.001
Antidepressant initiated before assessment	Not included	—	3.45 (2.68 to 4.22)	<0.001

Footnotes: Estimates were obtained from linear mixed-effects models incorporating participant-specific random intercepts. The analysis included 1,625 HDRS-17 observations from 480 participants with brain MRIs. Both models included follow-up time, baseline CSVD burden category, the CSVD burden × follow-up time interaction, age, baseline NIHSS score, and baseline HDRS-17 score. The sensitivity model additionally incorporated documented antidepressant initiation before each scheduled assessment as a time-varying covariate. Moderate-to-severe CSVD burden was defined as a Global CSVD Score of 2–4, with none-to-mild CSVD burden (score 0–1) as the reference category. The CSVD burden × follow-up time interaction indicates that longitudinal HDRS-17 trajectories differed between CSVD burden groups; the direction of this difference is illustrated by the model-predicted trajectories in Figure 3. Because antidepressant treatment was clinically assigned rather than randomized, and detailed information regarding adherence, medication switching, dose modification, and treatment continuity was not consistently available at every assessment, the antidepressant coefficient should not be interpreted as a causal treatment effect. The positive coefficient likely reflects, at least in part, confounding by indication, whereby participants with more severe depressive symptoms were more likely to initiate antidepressant treatment.

Abbreviations: CI, confidence interval; CSVD, cerebral small vessel disease; HDRS-17, 17-item Hamilton Depression Rating Scale; MRI, magnetic resonance imaging; NIHSS, National Institutes of Health Stroke Scale.
